# Beneficial Effects of Resveratrol-Mediated Inhibition of the mTOR Pathway in Spinal Cord Injury

**DOI:** 10.1155/2018/7513748

**Published:** 2018-03-26

**Authors:** Jingying Zhou, Xue Huo, Benson O. A. Botchway, Luyao Xu, Xiaofang Meng, Songou Zhang, Xuehong Liu

**Affiliations:** ^1^Department of Histology and Embryology, Medical College, Shaoxing University, Zhejiang, China; ^2^Institute of Neuroscience, Zhejiang University School of Medicine, Hangzhou, China

## Abstract

Spinal cord injury (SCI) causes a high rate of morbidity and disability. The clinical features of SCI are divided into acute, subacute, and chronic phases according to its pathophysiological events. The mammalian target of rapamycin (mTOR) signaling pathway plays an important role in cell death and inflammation in the acute phase and neuroregeneration in the subacute/chronic phases at different times. Resveratrol has the potential of regulating cell growth, proliferation, metabolism, and angiogenesis through the mTOR signaling pathway. Herein, we explicate the role of resveratrol in the repair of SCI through the inhibition of the mTOR signaling pathway. The inhibition of the mTOR pathway by resveratrol has the potential of serving as a neuronal restorative mechanism following SCI.

## 1. Introduction

Spinal cord injury (SCI) causes a high rate of morbidity and disability. Presently, more than 2.5 million people suffer from SCI, with annually reported new cases being about 12,000 in the United States [1, 2]. SCI is classified into primary and secondary phases. The primary phase of SCI begins when a sharp penetrating force lacerates or macerates the spinal cord. Additionally, a blunt force contusing or compressing the spinal cord falls under the primary phase of SCI. After primary injury, the injured area of the spinal cord increases gradually. This is when secondary injury takes place. Secondary injury includes events such as vascular disorder, glutamate excitotoxicity, apoptosis, and inflammation. Trauma engenders mechanical damages to sensitive capillaries and causes bleeding. Ischemia has been correlated with hemorrhages [3]. Disruption of the blood-spinal cord barrier leads to inflammatory cell infiltration. Vascular disorder triggers microglia to produce proinflammatory cytokines, followed by progressive cellular necrosis and the release of ATP, DNA, and K^+^ [4]. SCI creates a cytotoxic postinjury environment and activates microglia to recruit phagocytes. Neutrophils, monocytes, and microglia are the inflammatory cells involved in SCI [5]. Following SCI, neutrophils tend to increase in the primary lesion, producing oxidative and proteolytic enzymes. Macrophages release proinflammatory cytokines, nitric oxide, and proteases. Furthermore, glutamate excitotoxicity, which is a result of the release of glutamate and astrocytes, leads to further neuronal cell death [6]. Additionally, after SCI, the death of groups of neurons as well as microglia, oligodendrocytes, and astrocytes takes place. In white matter tracts, oligodendrocyte death lasts several weeks after SCI [7]. Oligodendrocyte death is advantageous to postinjury demyelination. Secondary injury lasts for several weeks, which in turn provides a therapeutic time window. Its effectiveness lies in reducing the destruction of the neural tissue during the mitigation of the above process. SCI treatment mainly includes surgical treatment during the early stage. During middle and late stages of SCI, drug interventions are employed. It is worth noting that all these stages possess certain therapeutic effects. In our precursory study, curcumin was found to have the capability of serving as a future therapeutic for SCI [8]. It is worth noting that one of the chief targets in SCI treatment is to improve the microenvironment and promote the regeneration of the injured site. Thus, in another study, we also deduced that subsequent to SCI, treatment with olfactory ensheathing cell- (OEC-) seeded poly(lactic-co-glycolic acid) (PLGA) complex could not only ameliorate the microenvironment but also promote cell differentiation [9]. In recent years, coupled with in-depth knowledge of the various signaling pathways, SCI treatment has been improved by interfering the procedure with various signaling pathways such as nuclear factor-kappa B (NF-*κ*B), mTOR, and mitogen-activated protein kinase (MAPK) [10–12]. The mTOR signaling pathway plays a very important role in the progress of cell death, inflammation, neuroregeneration, and regulation of glial scar following SCI.

## 2. Relationship between the mTOR Signaling Pathway and SCI

As a member of the phosphatidylinositol-3-kinase-related kinase superfamily, mTOR is a serine/threonine protein kinase. The combination of mTOR complex 1 (mTORC1) and mTOR complex 2 (mTORC2) protein complexes forms the mTORC signaling pathway. mTORC1 phosphorylates downstream effectors such as p70 ribosomal S6 protein kinase (p70S6K) and further regulates mRNA translation. Thus, mTORC1 is important in stimulating protein synthesis. mTORC2, on the other hand, has been demonstrated to phosphorylate members of the AGC kinase family including protein kinase B (Akt), which is linked to several pathological conditions. mTORC2 is regarded as a regulator of the actin cytoskeleton [13]. Phosphatidylinositol-3-kinase/protein kinase B (PI3K/Akt) is one of the major pathways that activate mTOR. mTOR plays an important role in several physiological functions in the CNS, which includes the regulation of neuronal cell growth and survival and development of axon and dendrite [14, 15]. Additionally, a number of pathophysiological diseases such as neurodegenerative cancer, cardiovascular cancer, and renal cancer have been correlated with the regulation of the mTOR pathway [16–19]. Studies pertaining to the function of mTOR in SCI have been reported to be hinging on the time phase following SCI [20].

### 2.1. Relationship between the mTOR Signaling Pathway and Acute/Subacute Stages of SCI

Regarding the acute phase in the wake of SCI, the mTOR signaling pathway participates in the regulation of cell death, activation of macrophage/microglia, and inflammation [20].

#### 2.1.1. mTOR Signaling Pathway Participates in Cell Death

Neuronal death includes neuronal apoptosis, autophagy, and necrosis. As mTOR's inhibitor, rapamycin could block the activation of the Akt/mTOR pathway, thereby preventing apoptosis of nerve cells [21]. Akt could elevate cyclin D1 by inactivating glycogen synthase kinase-3*β* (GSK-3*β*) and reducing protein 27 kinase inhibition protein 1 (p27). The inhibition of Akt phosphorylation leads to G1 arrest, which in turn induces apoptosis [22]. Also, the inhibition of the PI3K/Akt/mTOR signaling pathway could reduce the apoptosis-related proteins through the mitochondrial pathway after SCI [23]. TORC1 inhibition could hamper protein translation, resulting in the decrement of the Bcl-2 family and instigation of apoptosis [24]. Moreover, the inhibition of the PI3K/Akt/mTOR pathway could increase the expression of Beclin-1 [25]. A study discovered the augmentation of Beclin-1 expression to increase and decrease Bcl-2 and Bax expressions, respectively, eventually culminating in abated levels of apoptosis [26]. Autophagy precedes apoptosis, demonstrating a significant effect in regulating cell death [27]. Autophagy, a significant moderator, participates in pathological changes after SCI [28]. Autophagy is important to secondary injury's repair. The disruption of autophagy after SCI aggravates endoplasmic reticulum (ER) stress and causes cell death [29]. The inhibition of the mTOR signaling pathway could trigger autophagy [30]. Rapamycin induces the occurrence of autophagy by inhibiting the mTOR signaling pathway. Research studies have evidenced the employment of rapamycin to curtail the phosphorylation of p70S6K protein whilst augmenting the expression levels of LC3 and Beclin-1 [31]. Autophagy, as an intracellular catabolic mechanism, increases cell survival rate by degrading and recycling damaged organelles and inept proteins to provide ATP and amino acids [32, 33]. However, autophagy leads to cell death in certain pathological situations. Activating autophagy destroys injured cells and prevents neuronal loss. Studies found the activation of autophagy through the autophagosomal-lysosomal pathway to induce neuroprotective effects [34]. Autophagy starts off with the formation of autophagosomes that fuse with lysosomes to allow lysosomal hydrolases to degrade contents [35]. Autophagy flux is the progress including sequestration in autophagosome delivery and degradation in lysosomes [36]. Again, when neurons are injured, astrocytes are activated. Studies have reported the occurrence of apoptosis in astrocytes, which culminates in cell death during the early period of injury, subsequently curtailing the release of injurious factors [34]. This, ultimately, is adverse to neuronal regeneration. Additionally, there are reports suggesting a strong biochemical crosstalk mechanism between apoptosis and autophagy [37]. Both autophagy and apoptosis are potentially influenced by several similar signaling pathways such as p53, Ser/Thr kinases, and Bcl-2-homology-3-only proteins [38]. The cross-regulation of apoptosis and autophagy was demonstrated by suppressing each other. It is vital to point out that mitophagy (autophagy of mitochondria) is a key point in the inhibition of the apoptosis by autophagy in several maladies [39]. Hypoxia or ATP depletion is triggered by mitochondrial dysfunction, which in turn causes cytochrome release and the activation of caspase-9, and eventually, apoptosis.

#### 2.1.2. mTOR Signaling Pathway Regulates Inflammation

The traumatic spinal cord induces local inflammatory response by activated microglia, infiltrating neutrophils, and macrophages. Moreover, the upregulation of associated proinflammatory cytokines' expression has been reported to be induced by the traumatic spinal cord [40]. mTOR regulates the maturation of antigen-presenting cells such as T and B cells [41]. In a carried-out study, the mTOR pathway was found to improve the survival of EOC2 microglia in the oxygen-glucose deprivation stage whilst enhancing the expression of nitric oxide synthase 2 (NOS2) in the hypoxia stage in the BV2 microglial cell line. This suggests that mTOR participates in microglial proinflammatory activation, activates mTOR/MEK1/ERK1/2/IKK*β*/I*κ*B-*α*/NF-*κ*B, and results in inflammation [42, 43]. mTOR inhibition could improve anti-inflammation through the regulation of T cells [44]. Again, studies have evinced the inhibition of mTOR to control the activation of macrophage/microglia and curtail neuroinflammation [45]. mTOR inhibition could also reduce the proinflammatory cytokines produced by macrophages [46]. Furthermore, mTOR inhibition could bring about anti-inflammation via autophagy [47]. The instigation of autophagy could be as a result of impediment of mTOR. Autophagy, in turn, could degrade inflammasome components and remove the endogenous signals of inflammasome activation, thereby hindering inflammation. In addition, studies have evidenced rapamycin and temsirolimus to dramatically abridge the expressions of inducible NO synthase (iNOS), cyclooxygenase 2 (COX2), and glial fibrillary acidic protein (GFAP) and reestablish nNOS levels. Other researches have also demonstrated that mTOR inhibitors could accommodate the neuroinflammation in SCI [48]. Thus, inhibition of mTOR could decrease the inflammation procedure after SCI.

### 2.2. Relationship between the mTOR Signaling Pathway and the Chronic Phase of SCI

During the chronic phase following SCI, mTOR participates in regulating neuroregeneration and glial scar formation.

#### 2.2.1. mTOR Signaling Pathway Participates in Regulating Neuroregeneration

Injured neurons of the CNS undergo normal cell apoptosis rather than regeneration. The chief cause of central nerve regeneration failure has to do with inhibitory factors in the myelin as well as with the formation of glial scar and weak growth capability of mature neurons [49]. There are evidences explicating that mRNA and ribosomes of axons could take part in synthesizing cytoskeletal proteins [50, 51]. Researches have showed that the synthesis of axon local protein might participate in axonal regeneration [52]. During the chronic phase, damaged neural tissue regeneration is regulated by the mTOR signaling pathway. Immunohistochemistry results have propounded the mTOR signaling pathway to be present in neurons of nociceptive-specific C-fiber at the level of dorsal root ganglion and spinal cord neurons of inner lamina II [53]. Also, mTOR inhibition is instrumental in axonal regeneration. In the wake of SCI, the inhibition of mTOR by rapamycin could promote axonal regeneration via the suppression of new protein synthesis and cell proliferation. Improvement in CNS myelination and oligodendrocyte differentiation has also been connected to mTOR [20]. S6K1 (ribosomal protein S6 kinase 1) is the important downstream protein of mTOR [54]. Research studies demonstrated that after SCI, hindering S6K1 could promote regeneration of both the corticospinal tract and axon counts at 8 weeks [55]. Additionally, phosphatase and tensin homolog on chromosome 10 (PTEN), a lipid phosphatase, converts PIP3 to PIP2, subsequently inhibiting the downstream effectors of PI3K's activation [56]. Through the deletion of PTEN, there was activation of the PI3K/mTOR pathway and regulation of cell growth as well as proliferation by initiating cap-dependent protein translation [57, 58]. In addition, PTEN deletion was found to be a contributory factor in corticospinal tract regeneration, subsequently giving the impression that the PTEN/Akt/mTOR pathway could regulate axonal growth [59]. In another research study, the knocking out of tuberous sclerosis complex 1 (TSC1) was demonstrated to reactivate mTOR, which in turn promoted axonal regeneration [60]. However, TSC deficiency caused insulin resistance and resulted in unfolded protein response (UPR). It also regulated endoplasmic reticulum (ER) stress [61]. Interestingly, UPR initiates apoptotic pathways in the event when cells are unable to adapt to a condition in perturbation [62]. In barring cytoskeletal protein synthesis, microtubule assembly is instrumental in axonal regeneration, which has been confirmed to be a key in the instigation of the growth cone. A study evinced autophagy to be vital in both microtubule stability and axonal regeneration following CNS injury [63].

#### 2.2.2. mTOR Signaling Pathway Participates in Regulating Glial Scar

Glial scar is another major barrier for regeneration; thus, overcoming this barrier would be significant to axonal regeneration [64]. Glial scar consists of reactive astrocytes and connective tissues. The main component of the extracellular matrix is chondroitin sulfate proteoglycan. More specifically, astrocytes become hypertrophic and proliferative and form an astrocytic-rich border to produce glial scar after SCI [65]. Glial fibrillary acidic protein (GFAP), vimentin, and nestin cause glial hypertrophy. Thus, limiting astrocytic responses provides a potential therapeutic regimen in the enhancement of functional recovery after SCI. Moreover, mTOR could participate in astrogliosis by increasing cascaded downstream proteins and activating astrocytes [66]. Thus, regulating mTOR is a key to curtail the scar formation. Research has evinced astrocytes to be upregulated by an epidermal growth factor (EGF). EGF could phosphorylate Akt, causing the activation of mTOR as an important pathway of astrocyte physiology [67]. Several mTOR upstream regulators are vital to astrocytes. Luan et al. demonstrated that the downregulation of PI3K/Akt/mTOR expression could inhibit the formation of glial scar [68]. PI3K/Akt/mTOR inhibition could attenuate the formation of glial scar. Also, PTEN could negatively regulate the PI3K/Akt/mTOR pathway, thus showing a great function in attenuating glial scar formation [69]. Pharmacological inhibition using the mTOR-selective drug, rapamycin, was found to decrease astrogliosis and reduce GFAP expression at the injured site [70]. Autogenous hypertrophy and reentry into the cell cycle engender reactive astrogliosis. The regulation of the cell cycle is important in curtailing scar formation. Rapamycin could modulate the cell cycle to inhibit astrocyte proliferation. Additionally, a study found that rapamycin could restrain the proliferation of astrocytes by decreasing the cell number in the S stage [71]. These findings have clinical implications as potential SCI therapeutic applications with the inhibition of the mTOR signaling pathway.

## 3. Resveratrol Repairs SCI by Inhibiting the mTOR Signaling Pathway

**Figure 1:**
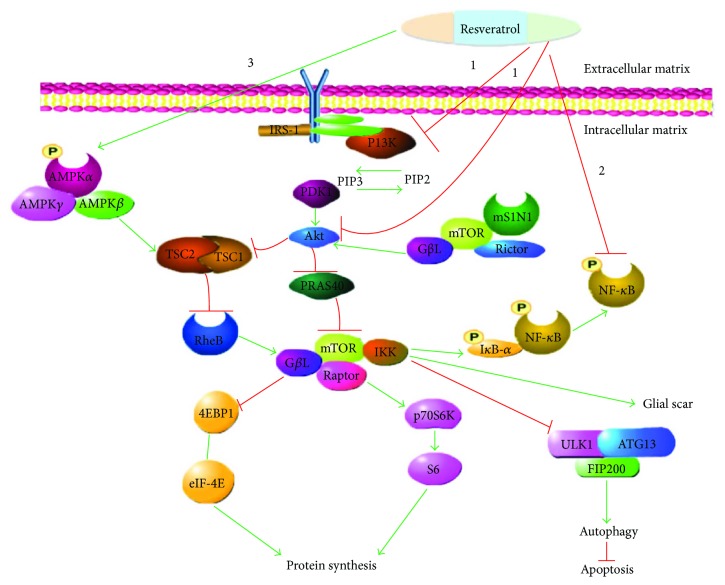
Resveratrol inhibits the mTOR signaling pathway in the repair of SCI. (1) Resveratrol can inhibit the PI3K and Akt and mTOR pathway so as to regulate protein synthesis, autophagy, and apoptosis. (2) mTORC1 can interact with IKK to connect with NF-*κ*B. (3) Resveratrol activates AMPK phosphorylation. mTOR, as a downstream signaling pathway of AMPK, can be inhibited by AMPK.

Each herb consists of numerous chemical constituents from different categories. Different active ingredients show therapeutic functions in a number of disease treatments. In recent years, traditional Chinese medicine (TCM) has been drawing attention in SCI treatment [72]. Resveratrol is a natural polyphenol antioxidant TCM. Its active ingredient is comprised of *Polygonum cuspidatum*, red grape skins, red wine, blueberries, and some nuts. Resveratrol has a number of biological activities and pharmacological actions, which include anti-inflammation, antioxidation, inhibition of platelet aggregation, and improvement of microcirculation [73–75]. Following neuronal injury, resveratrol exhibits its neuroprotective effects by regulating autophagy and apoptosis [76]. In the last few years, resveratrol has been explicated as a potential therapeutic in SCI treatment. Its therapeutic effect has been confirmed through behavioral scores [77]. Resveratrol can inhibit mTOR through several mechanisms such as PI3K and Akt [78, 79] (Figure 1). Both PI3K and Akt are mTOR upstream activators. Furthermore, resveratrol, in high concentration, has been evidenced to inhibit the mitochondrial function, decrease cellular ATP levels, and activate AMPK [80]. Moreover, the mTOR pathway could be inhibited by the activation of AMPK, as depicted in Figure 1. In a study conducted, expressions of AMPK and mTOR were increased and decreased, respectively, following resveratrol treatment [81]. Interestingly, resveratrol has also been evidenced to be involved in apoptosis, autophagy, and inflammation as well as in scar tissue improvement subsequent to SCI through the mTOR pathway; thus, resveratrol ameliorates SCI. Additionally, in the wake of SCI, resveratrol treatment improved the Bcl2/Bax ratio and decreased the expression level of caspase-3. What is more, research studies have also revealed the antiapoptotic effect of resveratrol [82]. The apoptotic effect of resveratrol was attributed to the inhibition of PI3K, Akt, and mTOR phosphorylation [83]. In a study by Zhao et al., the authors indicated augmentation in autophagy expression following resveratrol treatment in SCI [84]. In another study by Park et al., they posited that the suppression of mTOR activity might culminate in resveratrol instigating autophagy [85]. The detailed mechanism was through the inhibition of the mTOR-ULK1 pathway. mTOR inhibition reduced the hindrance of unc-51-like autophagy activating kinase 1 (ULK1) phosphorylation and induced autophagy. With that being said, autophagy is involved in neuroprotection [86]. Again, resveratrol has been evidenced to inhibit proliferation of pathological scar fibroblasts by decreasing mTOR expression and its downstream molecule p70S6K [87]. More so, resveratrol has been found to have an effect on inflammation. Treatment with resveratrol after SCI reduced the expression of inflammatory cytokines such as IL-1*β*, IL-10, and TNF-*α* [82]. Resveratrol has also been suggested to suppress NF-*κ*B's activity. mTOR and NF-*κ*B pathways are tied in many aspects. For instance, the activation of mTOR by Akt culminates in the activation of NF-*κ*B, which is associated with inhibitor of NF-*κ*B kinase (IKK) and mTORC1 complex's Raptor [80] (Figure 1). Suffice to say, resveratrol inhibits NF-*κ*B and inflammatory molecules through the mTOR pathway [88].

## 4. Conclusion

mTOR is involved in the regulation of several diseases, cellular functions, and trauma in the CNS. The mTOR signaling pathway plays an important role at different times. As such, the perspicacious knowledge concerning how the mTOR signaling pathway works in the process of neural protection is of great significance in SCI. TCM is an important supplementary treatment for SCI, which may offer therapeutic and reparative benefits in SCI. TCM might replace the application of nonsteroidal anti-inflammatory drugs, neurotrophic factors, or even methylprednisolone. Resveratrol participates in apoptosis, formation of pathological scar, and proliferation of fibroblasts as well as in anti-inflammation via the inhibition of mTOR in repairing SCI. Resveratrol, through the mTOR pathway, has the tendency of serving as an SCI therapeutic. However, further research pertaining to the effect and specific molecular mechanisms in the different phases of SCI still needs to be elucidated.

## Abbreviations

SCI: Spinal cord injury
mTOR: Mammalian target of rapamycin
OECs: Olfactory ensheathing cells
PLGA: Poly(lactic-co-glycolic acid)
NF-*κ*B: Nuclear factor-kappa B
MAPK: Mitogen-activated protein kinase
mTORC1: mTOR complex 1
mTORC2: mTOR complex 2
p70S6K: p70 ribosomal S6 protein kinase
Akt: Protein kinase B
P13K/Akt: Phosphatidylinositol-3-kinase/protein kinase B
GSK-3*β*: Glycogen synthase kinase-3*β*
P27: Protein 27 kinase inhibition protein 1
Bcl-2: B cell lymphoma-2
ER: Endoplasmic reticulum
NOS2: Nitric oxide synthase 2
COX2: Cyclooxygenase 2
iNOS: Inducible nitric oxide synthase
GFAP: Glial fibrillary acidic protein
S6K1: Ribosomal protein S6 kinase 1
PTEN: Phosphatase and tensin homolog on chromosome 10
TSC1: Tuberous sclerosis complex 1
UPR: Unfolded protein response
EGF: Epidermal growth factor
TCM: Traditional Chinese medicine
ULK1: unc-51-like autophagy activating kinase 1
IKK: Inhibitor of NF-*κ*B (nuclear factor *κ*B) kinase.
